# Microscopic analyses of weathered granite in ion-adsorption rare earth deposit of Jianxi Province, China

**DOI:** 10.1038/s41598-020-76981-8

**Published:** 2020-11-19

**Authors:** Hiroki Mukai, Yoshiaki Kon, Kenzo Sanematsu, Yoshio Takahashi, Motoo Ito

**Affiliations:** 1 National Institute of Advanced Industrial Science and Technology, 1-1-1 Higashi, Tsukuba, Ibaraki 305-8567 Japan; 2 Present Address: Faculty of Life and Environmental Sciences, University of Tsukuba, Tennodai 1-1-1, Tsukuba, Ibaraki 305-8572 Japan; 3 Department of Earth and Planetary Science, Graduate School of Sciences, The University of Tokyo, 7-3-1 Hongo, Bunkyo-ku, Tokyo, 113-0033 Japan; 4 Kochi Institute for Core Sample Research, Japan Agency for Marine-Earth Science and Technology (JAMSTEC), Monobe B200, Nankoku, Kochi 783-8502 Japan

**Keywords:** Geochemistry, Mineralogy, Economic geology

## Abstract

Weathered granite of ion-adsorption rare earth elements (REEs) ore collected at Jiangxi Province, China was investigated to identify the minerals abundant in REEs. The analyses of scanning electron microscopy (SEM)-energy dispersive spectrometry (EDS) and laser ablation inductively coupled plasma mass spectrometry (LA-ICP-MS) for individual mineral particles of the weathered granite showed that kaolinitic particles formed by K-feldspar weathering contained large amounts of REEs. Scanning transmission electron microscopy (STEM)-EDS analyses revealed that the kaolinitic particles were mainly composed of kaolinite, illite and hematite. The elemental maps by Nano-SIMS for the kaolinitic particle clarified that La and Y are particularly concentrated in illite. The presence of illite presumably contributes to the formation of the REE accumulation zone in weathered granite. Furthermore, in the in-situ desorption experiment, nearly half the REEs (45.5%) remained in the kaolinitic particle after the treatment with 0.5 M ammonium sulfate solution. The desorption ratio of heavy REEs (HREEs: Gd–Lu) (60.4%) was lower than that of light REEs (LREEs: La–Eu) apart from Ce (69.0%). These results suggest that REEs form inner-sphere complexes on the kaolinitic particle. It can be assumed that the inner-sphere complexation suppresses the extraction ratio of REEs from the ores by ion-exchange treatment.

## Introduction

Presently, China constitutes the majority of rare earth elements (REEs: La–Lu) production, which is essential for cutting edge industries worldwide^1^. Among the ores for REE production, ion-adsorption type ores existing mainly in Mesozoic granitic rocks of southern China are an important source of heavy REEs (HREEs: Gd–Lu), which are mostly more precious than light REEs (LREEs: La–Eu)^2^. This type of ore typically contains more than 50% REEs that can be extracted with ion-exchange treatments, such as ammonium sulfate [(NH_4_)_2_SO_4_] solution^3–8^. Moreover, the ores have less radioactive elements (U and Th) than the other REE ores such as carbonatite and placer heavy minerals^9^. Owing to these advantages, this deposit type is minable. However, the concentration of REEs in the ores is relatively low (< 2000 ppm)^4,8,10^, compared to the other types of REE ores, such as carbonatite (approx. 0.5–10 wt%) and alkaline rock types (approx. 0.5–2 wt%)^2,11–13^.

The ion-adsorption REE deposits are formed by weathering of underlying parent granite, and their weathering profiles can be separated into four units: (A) surface soil zone, (B) wholly weathered zone, (C) sub-weathered zone, and (D) slightly weathered zone^3,4^. In the upper layer, the content of clay (kaolinite and halloysite) increases due to the weathering of feldspar^3,4,14,15^. It has been reported that REEs are concentrated in the second and third layers (B and C)^4,10^. The weathering of the parent granite involves the alteration of REE-bearing primary minerals, such as allanite, titanite, fluorocarbonates (synchysite, parasite, and bastnaesite), gadolinite, hingganite, and yttrialite^3,8,10,16^. The dissolved REEs from the primary minerals migrate downward into the weathered granite profile and REEs adsorbed on clays of the aforementioned layers. Fractionation between Ce and other REEs has been observed in many weathering profiles^17–19^. The Ce anomaly can be attributed to the oxidation of Ce^3+^ to Ce^4+^, wherein Ce^4+^ is less insoluble in water under ambient conditions. The occurrence of REE-bearing minerals resistant to weathering, such as zircon, monazite, and xenotime, reduce the REE extraction ratio by ion-exchange treatment and presumably influence the LREEs/HREEs ratio.

In ion-adsorption ores, it has been estimated that clay minerals adsorb REEs, particularly the kaolin mineral group (kaolinite and halloysite)^3,20–23^. These clay minerals commonly occur in the ion-adsorption ores and have a low point of zero charge (PZC) (< 4.5 in most cases)^24,25^. The 1:1 layer clay minerals have a small permanent charge derived from isomorphous substitution (e.g., Al^3+^ for Si^4+^)^24^, in addition to a surface charge that is mainly derived from the variable pH-dependent charge. The REEs adsorbed on the surface of the clays, forming outer-sphere complexes, are assumed to be extracted by ion-exchange treatment. However, direct analyses of individual minerals have rarely been conducted because of the low concentration of REEs in the ores. Even after the ion-exchange treatment, nearly half the REEs still remain in the weathered granite^7^. The detailed distributions and chemical states of REEs in individual minerals are still unclear. Understanding the REEs behavior in weathered granite is also important for considering the geological disposal of high-level radioactive wastes. Granitic sites can be potential candidates for such disposals, and, among REEs, Eu-(III) has been used as an analog of trivalent actinides such as Am-(III) or Cm-(III)^26–30^.

In our study, individual mineral particles constituting weathered granite collected from an ion-adsorption deposit in China were characterized using scanning and transmission electron microscopy (SEM and TEM). For the elemental analyses of REEs in the mineral particles, laser ablation inductively coupled plasma mass spectrometry (LA-ICP-MS) and nano-scale secondary ion mass spectrometry (Nano-SIMS) were combined with electron microscopic analyses to overcome the detection limit of the energy dispersive X-ray spectrometry (EDS). Through the combined analyses, we attempted to clarify the detailed distribution of REEs in an ion-adsorption ore and identify the minerals abundant in REEs. Furthermore, the chemical states of REEs in the mineral particles were also investigated through in-situ desorption experiments with ion-exchange treatments.

## Results

### XRD and SEM-EDS analyses of weathered granite

**Figure 1:**
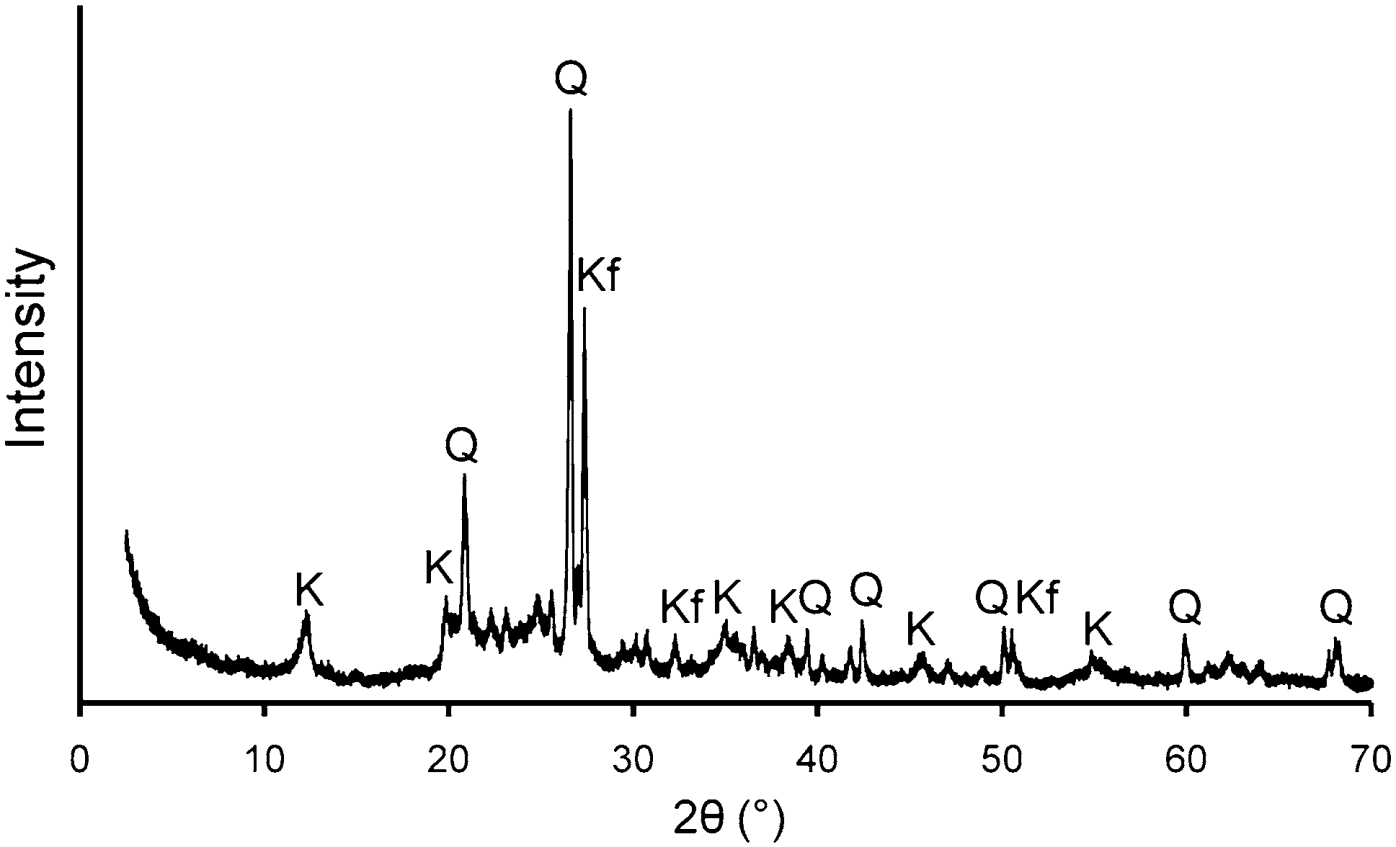
X-ray diffraction (XRD) pattern of weathered granite sample. The sample was collected from an ion-adsorption ore in Dingnan County, Jiangxi Province, China. The major peaks in the pattern were attributed to the following minerals: *Q* quartz, *Kf* K-feldspar, *K* Kaolinite.

**Figure 2:**
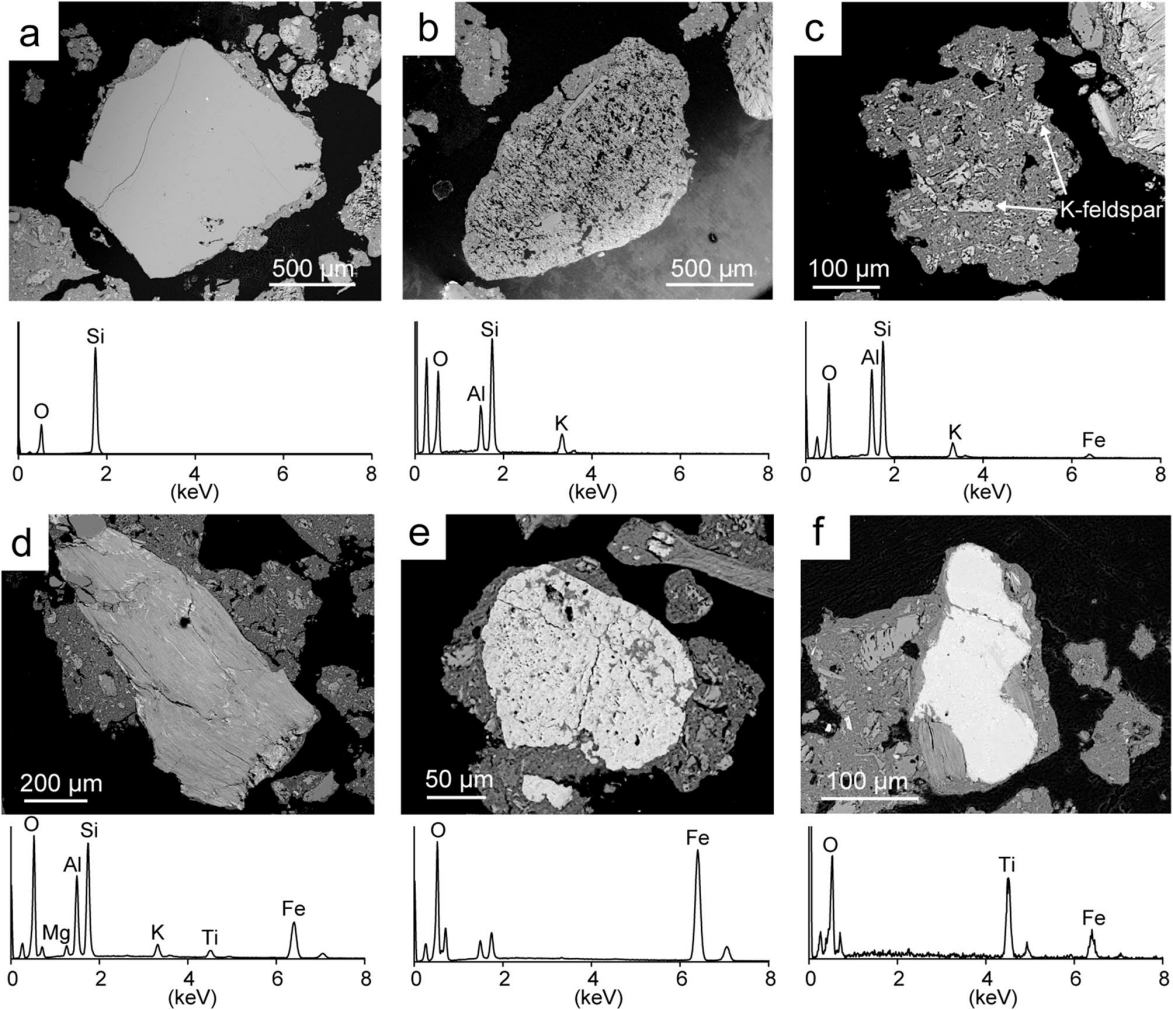
Back-scattered electron (BSE) images and energy dispersive X-ray spectrometry (EDS) spectra of mineral particles in weathered granite; (**a**) quartz; (**b**) K-feldspar; (**c**) kaolinitic particle; (**d**) micaceous particle; (**e**) hematite; and (**f**) ilmenite. (**a**–**c**) are major minerals, and (**d**–**e**) are minor minerals in the weathered granite.

Results of X-ray diffraction (XRD) measurements indicated that weathered granite, collected from an ion-adsorption ore in Dingnan County, Jiangxi Province, China, is mainly composed of quartz, K-feldspar, and kaolinite (Fig. 1). SEM-EDS analyses of the mineral particles showed quartz, K-feldspar and kaolinitic particles, as indicated by the XRD results with minor amounts minerals of micaceous particles, hematite, and ilmenite (Fig. 2). As REE-bearing minerals, zircon and monazite were partly observed. Unlike pure kaolinite, the kaolinitic particles often contained K-feldspar fragments, which were formed by K-feldspar weathering, and the EDS spectrum showed peaks of K and Fe in addition to those of O, Al, and Si. Conversely, we found that the chemical composition of the micaceous particle had a lower concentration of K than that of fresh biotite. Such a chemical composition suggests that the micaceous particles were derived from biotite weathering^31,32^.

### REEs measurements of individual mineral particles by LA-ICP-MS

**Figure 3:**
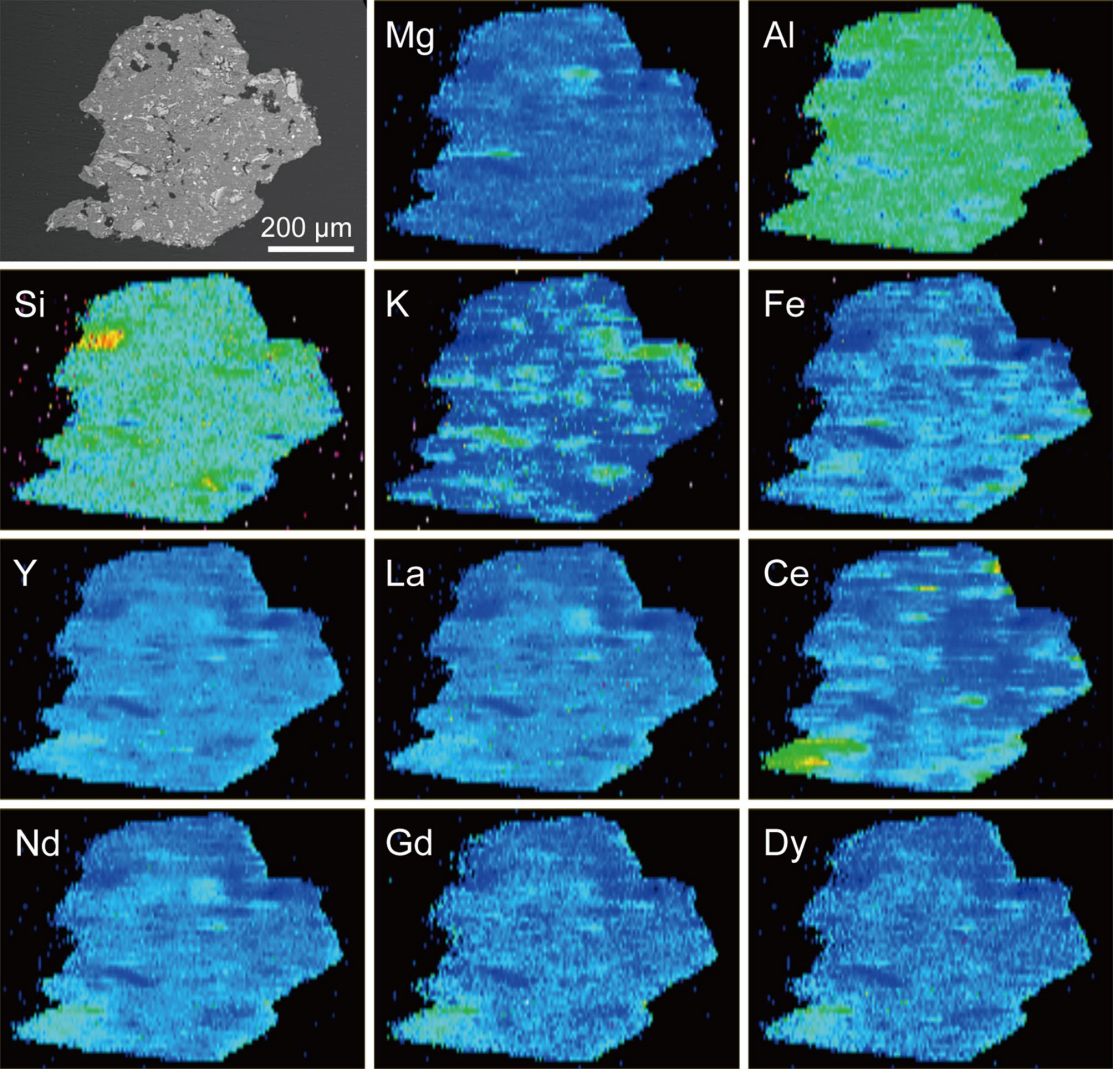
BSE image and elemental maps of Mg, Al, Si, K, Fe, Y, La, Ce, Nd, Gd, and Dy for a kaolinitic particle. The elemental mapping was performed using time-resolved analysis (TRA) mode by laser ablation inductively coupled plasma mass spectrometry (LA-ICP-MS).

The concentrations of REEs in the individual mineral particles were investigated by LA-ICP-MS measurements (Table 1). The total REE concentrations were as follows: quartz (13.2 ppm), K-feldspar (95.6 ppm), kaolinitic particles (519–1396 ppm), micaceous particles (543–1393 ppm), hematite (441 ppm), and ilmenite (115 ppm). For the kaolinitic and micaceous particles, measurements were conducted for six and five particles, respectively. K-feldspar fragments were avoided in the measurements of kaolinitic particles. The results indicate that the kaolinitic and micaceous particles are abundant with REEs compared to the bulk of the ion-adsorption ore (474 ppm) (Supplementary Table S1)^33^. Among the other minerals, hematite showed relatively high REE concentrations. Some of the kaolinitic particles contained high proportions of Ce and they showed both positive and negative Ce anomalies ([Ce/Ce*]_N_ = 0.25–6.64), although the bulk of the weathered granite showed negative Ce anomaly ([Ce/Ce*]_N_ = 0.64) (Supplementary Table S1)^33^. In contrast, micaceous particles showed negative Ce anomaly ([Ce/Ce*]_N_ = 0.03–0.22) in the analyses. The abundance of individual mineral particles, estimated from the analyses of XRD and SEM-EDS, suggests that REEs are mainly distributed in the kaolinitic particles in the weathered granite. Figure 3 shows the results of elemental maps for a kaolinitic particle analyzed by LA-ICP-MS. Each element was distributed heterogeneously in the particle. Some parts with high concentrations of K appeared to be associated with the fragments of K-feldspar, but K was also distributed in the other parts of the particle. The distribution of Fe is approximately inversely correlated to the parts with high concentrations of K. REEs were not distributed in the K-feldspar fragments, as presented in Table 1. REEs showed a similar distribution within the particle, although Ce had a different distribution than the others.

**Table 1:** The concentrations of Y and REEs in the individual mineral particles.

	Quartz	K-feldspar	Kaolinitic particle 01	Kaolinitic particle 02	Kaolinitic particle 03	Kaolinitic particle 04	Kaolinitic particle 05
Y (ppm)	7.34	11.5	74.3	97.5	72.5	64.4	72.6
La (ppm)	0.03	8.41	61.4	224	182	195	83.4
Ce (ppm)	2.42	66.9	629	773	30.5	24.7	262
Pr (ppm)	0.28	1.41	24.1	60.4	45.4	48.0	20.2
Nd (ppm)	2.73	6.53	97.1	203	161	163	76.2
Sm (ppm)	0.80	1.58	28.2	40.2	29.3	31.1	16.8
Eu (ppm)	0.75	2.72	5.41	13.1	9.42	9.76	6.53
Gd (ppm)	1.14	1.88	26.3	33.6	24.5	25.3	17.9
Tb (ppm)	0.23	0.32	4.04	4.22	3.18	3.01	2.62
Dy (ppm)	1.13	2.24	23.1	21.6	17.1	15.3	14.6
Ho (ppm)	0.23	0.37	4.04	3.70	3.10	2.60	2.63
Er (ppm)	1.15	1.13	11.7	9.54	7.56	6.89	7.76
Tm (ppm)	0.26	0.28	1.72	1.19	1.03	0.80	1.08
Yb (ppm)	1.88	1.51	12.4	7.86	6.53	5.35	6.69
Lu (ppm)	0.20	0.26	1.68	1.09	0.98	0.67	0.93
LREE (ppm)	7.02	87.6	845	1314	458	472	465
LREE^a^ (ppm)	4.59	20.6	216	541	427	447	203
HREE (ppm)	6.23	8.00	85.0	82.8	64.0	59.9	54.3
REE (ppm)	13.2	95.6	985	1397	522	532	519
REE^a^ (ppm)	10.8	28.7	301	624	491	507	257
REY (ppm)	20.6	107	1059	1494	594	596	592
LREE/HREE	1.13	10.9	9.94	15.9	7.15	7.88	8.57
LREE^a^/HREE	0.74	2.58	2.54	6.54	6.67	7.47	3.74
Y/Ho	32.3	30.9	18.4	26.3	23.4	24.7	27.6
[Ce/Ce*]_N_	5.94	4.66	2.43	6.64	0.34	0.25	1.53
[Eu/Eu*]_N_	2.38	4.80	0.20	0.36	0.35	0.35	1.14
[La/Yb]_N_	0.01	3.79	24.8	19.4	19.0	24.8	12.5

	Micaceous particle 01	Micaceous particle 02	Micaceous particle 03	Micaceous particle 04	Hematite	Ilmenite	
Y (ppm)	224	152	145	102	86.0	26.2	
La (ppm)	497	322	328	179	87.4	19.4	
Ce (ppm)	87.6	34.8	29.1	10.1	131	35.8	
Pr (ppm)	114	79.6	78.1	46.6	22.7	5.0	
Nd (ppm)	410	291	282	177	89.4	15.9	
Sm (ppm)	80.3	53.8	54.3	35.9	21.9	4.27	
Eu (ppm)	21.9	15.7	16.8	5.20	7.17	0.93	
Gd (ppm)	64.2	42.9	46.7	31.4	21.5	3.7	
Tb (ppm)	9.28	5.65	6.20	4.26	3.32	0.99	
Dy (ppm)	50.4	31.7	33.4	22.7	20.5	7.92	
Ho (ppm)	8.86	5.30	6.22	4.06	4.09	1.86	
Er (ppm)	23.6	13.2	16.5	11.9	13.1	7.1	
Tm (ppm)	3.21	1.80	2.40	1.60	2.18	1.24	
Yb (ppm)	19.7	11.5	15.7	11.5	14.8	9.22	
Lu (ppm)	2.97	1.62	2.05	1.59	2.16	1.45	
LREE (ppm)	1211	797	788	454	359	81.2	
LREE^a^ (ppm)	1123	762	759	444	229	45.5	
HREE (ppm)	182	114	129	89.1	81.7	33.5	
REE (ppm)	1393	911	917	543	441	115	
REE^a^ (ppm)	1305	876	888	533	310	79.0	
REY (ppm)	1617	1063	1063	645	527	141	
LREE/HREE	6.64	7.01	6.10	5.10	4.40	2.42	
LREE^a^/HREE	6.16	6.70	5.87	4.98	2.80	1.36	
Y/Ho	25.3	28.7	23.4	25.0	21.0	14.1	
[Ce/Ce*]_N_	0.09	0.22	0.18	0.03	0.70	0.87	
[Eu/Eu*]_N_	0.93	0.33	0.33	0.47	1.00	0.71	
[La/Yb]_N_	17.2	19.1	14.2	10.6	4.01	1.43	

^a^Total concentration values excluding Ce.[Ce/Ce*]_N_: Ce_N_/(La_N_ × Pr_N_)^1/2^, [Eu/Eu*]_N_: Eu_N_/(Sm_N_ × Gd_N_)^1/2^, [La/Yb]_N_: La_N_/Yb_N_ [where N is normalized by C1-chondrite^35^.

### In-situ desorption experiments of REEs in clay mineral particles

In-situ desorption experiments of REEs with ammonium sulfate solution were applied to the kaolinitic and micaceous particles [described as Kaolinitic particle 01 (K01) and Micaceous particle 01 (M01) in Table 1] (Table 2). The desorption ratios of REEs in total were 54.5% and 79.2% (except for Ce: 66.6% and 79.7%) in K01 and M01, respectively. REEs desorbed better from M01 than from K01. From the results, it can be understood that REEs remained in the clay mineral particles after the ion-exchange treatment. The relatively low desorption ratios of Ce (K01: 48.5%, M01: 70.8%) can be attributed to the presence of the Ce^4+^ mineral, cerianite [(Ce^4+^, Th)O_2_], which is commonly formed at oxidized conditions near surface. Fractionation between LREEs (excluding Ce) and HREEs were also observed in the particles. The desorption ratios of HREE (K01: 60.4%, M01: 65.1%) were lower than those of LREE excluding Ce (K01: 69.0%, M01: 82.1%). Fractionation was stronger in M01 than in K01. Reductions in the Y/Ho ratio were also commonly found in the particles.

**Table 2:** Results of the in-situ desorption experiments in the Kaolinitic particle 01 and the Micaceous particle 01.

Kaolinitic particle01				Micaceous particle01			
	Before tretment	After treatment	Desorption ratio (%)		Before tretment	After treatment	Desorption ratio (%)
Y (ppm)	74.3	23.6	68.2	Y (ppm)	224	66.4	70.3
La (ppm)	61.4	16.2	73.6	La (ppm)	497	84.4	83.0
Ce (ppm)	629	324	48.5	Ce (ppm)	87.6	25.6	70.8
Pr (ppm)	24.1	7.30	69.8	Pr (ppm)	114	21.2	81.3
Nd (ppm)	97.1	32.4	66.6	Nd (ppm)	410	72.4	82.4
Sm (ppm)	28.2	9.15	67.6	Sm (ppm)	80.3	18.2	77.3
Eu (ppm)	5.41	2.00	63.1	Eu (ppm)	21.9	5.25	76.0
Gd (ppm)	26.3	8.08	69.2	Gd (ppm)	64.2	15.1	76.4
Tb (ppm)	4.04	1.45	64.2	Tb (ppm)	9.28	2.70	70.9
Dy (ppm)	23.1	10.2	55.6	Dy (ppm)	50.4	17.1	66.2
Ho (ppm)	4.04	1.64	59.5	Ho (ppm)	8.86	3.40	61.7
Er (ppm)	11.7	4.88	58.4	Er (ppm)	23.6	10.3	56.2
Tm (ppm)	1.72	0.74	56.9	Tm (ppm)	3.21	1.62	49.7
Yb (ppm)	12.4	5.73	53.9	Yb (ppm)	19.7	11.7	40.7
Lu (ppm)	1.68	0.93	45.0	Lu (ppm)	2.97	1.74	41.3
LREE (ppm)	845	391	53.7	LREE (ppm)	1211	227	81.2
LREE^a^ (ppm)	216	67.1	69.0	LREE^a^ (ppm)	1123	201	82.1
HREE (ppm)	85.0	33.7	60.4	HREE (ppm)	182	64	65.1
REE (ppm)	985	448	54.5	REE (ppm)	1393	291	79.1
REE^a^ (ppm)	301	101	66.6	REE^a^ (ppm)	1305	265	79.7
REY (ppm)	1059	472	55.4	REY (ppm)	1617	357	77.9
LREE/HREE	9.94	11.6		LREE/HREE	6.64	3.57	
LREE^a^/HREE	2.54	1.99		LREE^a^/HREE	6.16	3.16	
Y/Ho	18.4	14.4		Y/Ho	25.3	19.6	
[Ce/Ce*]_N_	2.43	4.43		[Ce/Ce*]_N_	0.09	0.14	
[Eu/Eu*]_N_	0.20	0.20		[Eu/Eu*]_N_	0.93	0.96	
[La/Yb]_N_	24.8	11.9		[La/Yb]_N_	17.2	4.92	

### Detailed mineralogical characterization of clay mineral particles in TEM

**Figure 4:**
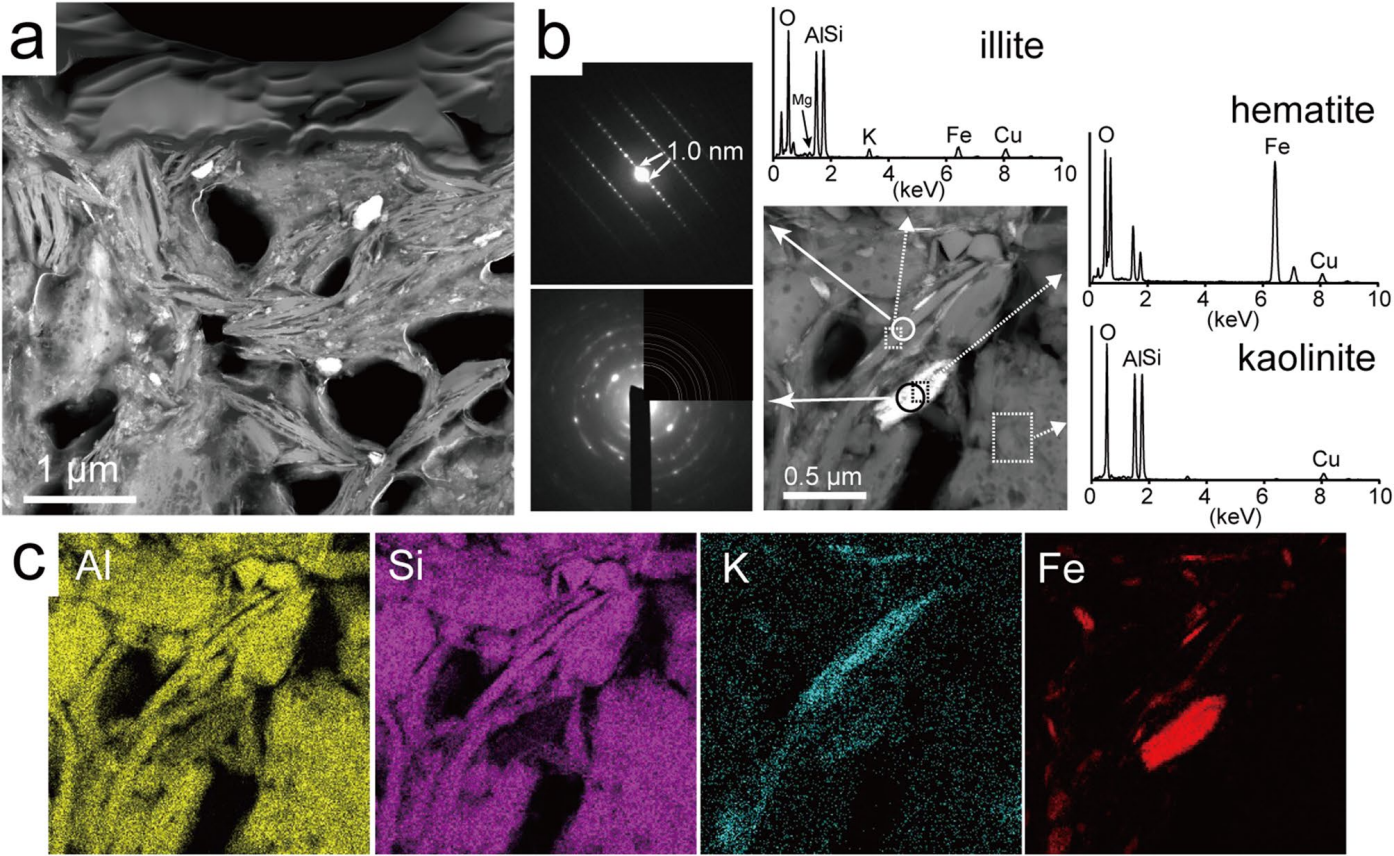
Results of the analyses of the kaolinitic particles by transmission electron microscopy (TEM). (**a**) High-angle annular dark field (HAADF)-scanning transmission electron microscopy (STEM) image of a kaolinitic particle. (**b**) Magnified HAADF-STEM image, electron diffraction patterns, and EDS spectra of the kaolinitic particle. The diffraction patterns and the spectra were obtained as circles and rectangles, respectively. The upper right part of the lower electron diffraction image is the simulated pattern of hematite. (**c**) STEM-EDS elemental maps of Al, Si, K and Fe measured in the HAADF-STEM image in (**b**).

**Figure 5:**
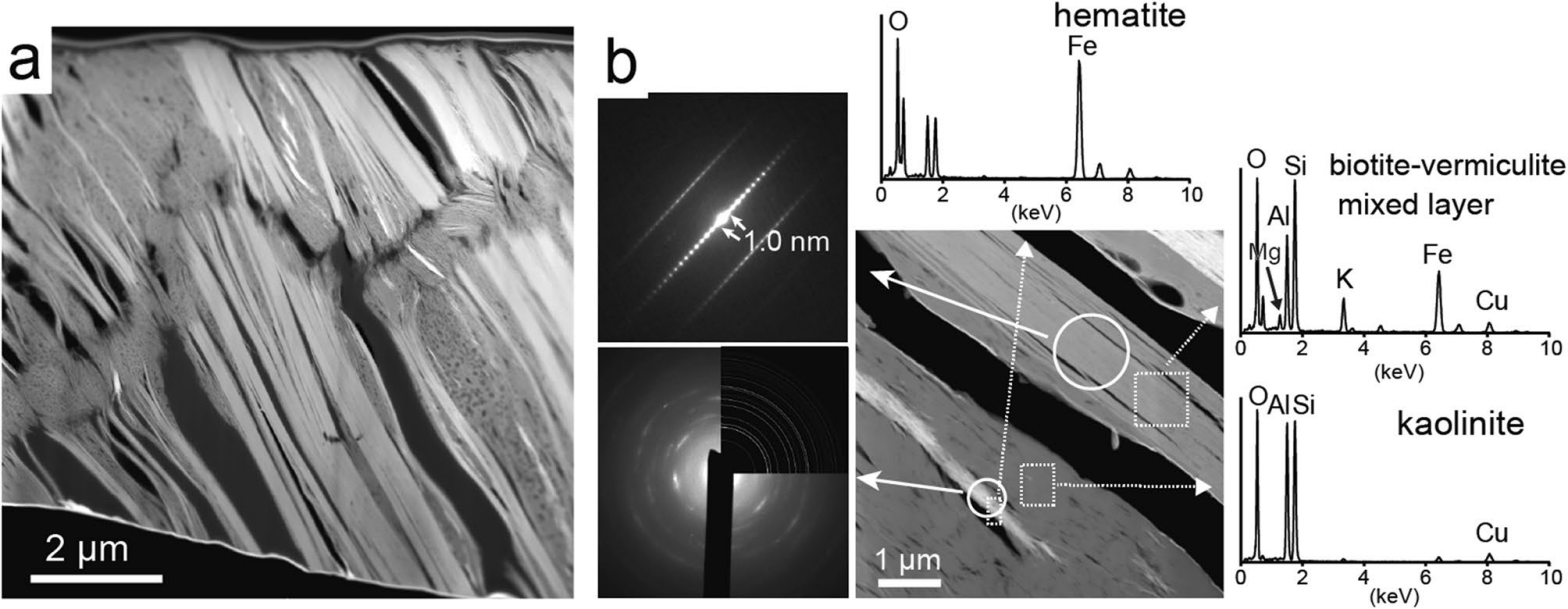
Results of the analyses for the micaceous particles in TEM. (**a**) Cross-sectional HAADF-STEM image of a micaceous particle. (**b**) HAADF-STEM image, electron diffraction patterns, and EDS spectra of a micaceous particle. The diffraction patterns and the spectra were obtained as circles and rectangles, respectively. The upper right part of the lower electron diffraction image is the simulated pattern of hematite.

Several thin sections of kaolinitic and micaceous particles were mineralogically characterized by TEM analyses. From the kaolinitic particle, the thin sections were processed avoiding the K-feldspar fragments. The HAADF-STEM image shows that the kaolinitic particle has irregularly laminated structures (Fig. 4a). STEM-EDS analyses and electron diffraction patterns revealed that the laminated structures were mainly composed of kaolinite and illite (Fig. 4b). Fine particles of iron oxide, identified as hematite from the electron diffraction pattern, were distributed at the surface of the structures. STEM-EDS elemental maps suggested that the parts concentrated with K, representing the illite, are relatively small fractions in the laminations (Fig. 4c). Cerianite was also found to be partly distributed in the kaolinitic particles (Supplementary Fig. S1). On the other hand, a cross-sectional HAADF-STEM image of the micaceous particle shows two main kinds of contrast (dark and bright parts) in the developed cleavage structures (Fig. 5a). STEM-EDS analyses and the electron diffraction patterns indicated that the bright and dark parts were kaolinite and biotite, respectively (Fig. 5b). The biotite crystals are presumably biotite-vermiculite (B–V) mixed-layers minerals formed by weathering^31,34^. The thin crystals of hematite were also partly observed along the cleavage direction.

### Elemental maps of clay mineral particles by Nano-SIMS

**Figure 6:**
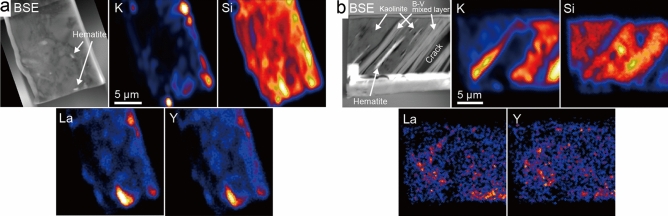
BSE images and elemental maps of K, Si, La, and Y for focused ion beam (FIB) sections. The elemental imaging with high spatial resolution and sensitivity was performed for FIB sections of (**a**) the kaolinitic particle and (**b**) the micaceous particle by nano-scale secondary ion mass spectrometry (Nano-SIMS).

Lastly, elemental mapping of K, Si, La, and Y was performed for the focused ion beam (FIB) sections of kaolinitic and micaceous particles by Nano-SIMS (Fig. 6a,b). In the back-scattered electron (BSE) image of the kaolinitic particle, the results of STEM-EDS analyses indicate that the bright parts are hematite while the other parts are kaolinite or illite. With the help of elemental maps, parts concentrated with K can be attributed to the presence of illite. La and Y are roughly concentrated in the illite, although they are also weakly distributed in the kaolinite. The distributions of La and Y are approximately the same. Since the FIB section was processed avoiding the K-feldspar fragments, the distributions of K and REEs appears different between LA-ICP-MS and Nano-SIMS measurements. On the other hand, in the BSE image of the micaceous particle, the brightest thin crystal is hematite. It is assumed that the darker and brighter parts are kaolinite and the B–V mixed layer, respectively. In the elemental map, parts concentrated with K represent the B–V mixed layer. The elemental maps of La and Y did not show significant differences in contrast, unlike the kaolinitic particle. However, they are not distributed significantly in the upper left part of the kaolinite. It appears that La and Y are distributed rather in parts of the B–V mixed layer.

## Discussion

The in-situ desorption experiments for the clay mineral particles showed that some REEs still remained in the clay minerals after the treatment with the ammonium sulfate solution. The results of the experiments suggest that REEs form inner-sphere complexes on the clay mineral particles. The fractionation between LREEs and HREEs can be attributed to the lanthanide contraction. It can be assumed that HREEs, with smaller ionic radii, are more strongly sorbed in the inner-sphere complexations than LREEs^36^. Since the ionic radii of Y and Ho are slightly different, the Y/Ho ratio can change from their original value through chemical reactions^37–39^. It is considered that the changes in the ratio represent the differences in the local structures between the cations in the particles. Under low REE concentrations, the formation of inner-sphere complexes is presumably predominant than the outer-sphere complexes on the clay minerals. Alternatively, the inner-sphere complexes more tightly sorbed on the clay minerals were likely to remain for the long period of ore formation. The low desorption ratios of Ce can be attributed to cerianite. From the bulk sample, HREEs (64.4%) desorbed better than LREEs (51.6%)^33^, in contrast to clay mineral particles. The SEM-EDS analyses suggest that the monazite, a LREE-bearing mineral, suppressed the desorption of LREEs during the bulk weathered granite treatments. The inner-sphere complexation on the clay mineral particles, in addition to REE-bearing minerals, presumably contributed to the decrease in REE desorption ratios during the cation exchange treatment of the ion-adsorption ores.

The abundance of the mineral particles indicated that REEs were mainly distributed to the kaolinitic particles, in addition to residual REE-bearing minerals such as zircon and monazite, in the weathered granite. The results of XRD measurements suggested that kaolinite is more abundant than other clay minerals. Thus, kaolinite in the particles possibly plays a key role in the behavior of REEs in the ore, as suggested in previous studies^3,4^. However, the elemental maps by Nano-SIMS for the kaolinitic particle revealed that REEs are particularly concentrated in illite. Illite has a low PZC (< 2)^25^ and basal plane permanent charge. In 2:1 layer clay minerals, the permanent charge plays a major role in the surface charge and provides sorption sites for REEs in the interlayers, unlike the 1:1 layer clay minerals^26^. Sorption experiments of Eu-(III) have suggested that illite has weak and strong sites situated along the edges of the clay platelets with amphoteric surface hydroxyl groups, in addition to the cation exchange sites^40,41^. These weak and strong sites are known to have higher selectivity for trace metals with low capacity and cause surface complexation reactions, which are regarded as inner-sphere complexation. It has been reported that kaolinite can form inner-sphere complexes for REEs at the edges, in addition to outer-sphere complexes^36,42^. However, the results of the elemental maps suggest that these sites of illite have higher affinity and/or larger site capacities for REEs than those of surrounding kaolinite under the conditions of the weathered granite (e.g., pH, ionic strength). In the in-situ desorption experiments, REEs in the outer-sphere complexes must have been desorbed by the ion-exchange treatments and some amounts of the REEs in the inner-sphere complexes were probably also desorbed, although REEs still remained after the ion-exchange treatments. Considering the site types of illite^40,41^, it can be hypothesized that REEs were mainly desorbed from the weak sites and remained in the strong sites. Moreover, the REEs sorbed onto kaolinite, which may form inner-sphere complexes, possibly also contribute to the desorption.

In the micaceous minerals, mainly composed of kaolinite and the B–V mixed layer, slight differences in Y and La concentrations between the mineral phases were observed in the elemental maps. Under low concentrations, many studies have reported that vermiculite has a superior adsorption property for cesium at frayed edge sites such as those in illite^34,43^. Although considering the difference between the monovalent and trivalent cations is required, it is possible that vermiculite has a high affinity for REEs forming inner-sphere complexes and the presence ratio of vermiculite in the micaceous particle influences the REE concentration. In the in-situ desorption experiments, some REEs might have been enclosed at the interlayer spaces because vermiculite can fix NH_4_^+^ or K^+^ by the collapse of the spaces. The variations in REEs concentrations in the clay mineral particles are presumably influenced by the mineral compositions of the individual particles and the behavior of the fluid containing REEs in weathered granite. The micaceous particles with a developed cleavage are considered to be able to contact a relatively large amount of fluid.

Hematite can have a high cation exchange capacity (CEC) depending on pH (< 100 meq/100 g)^44^. Rabung et al.^27^ suggested that hematite can form inner-sphere complexes for Eu (III). These properties possibly produced relatively high concentrations of REEs. The quartz and K-feldspar particles showed low REE concentrations. Their small CEC (1–2 meq/100 g) and surface areas, compared to clay minerals^45^, are consistent with the results of the measurements. K-feldspar will increase the REE concentration, depending on the degree of kaolinization. Halloysite and smectite were not observed in the analyzed sample, but can also be produced by the weathering of feldspar^14,15^. Halloysite, which is reported to be another abundant mineral in weathered granite, also has low PZC (< 2)^25^ and larger surface area due to tube-like structures compared to kaolinite. However, the atomic arrangements at the edges of the clay platelets should basically be the same as kaolinite. Thus, at low concentrations, the sorption ability of halloysite for REEs is presumably not very different from that of kaolinite. Smectite has high CEC (70–120 meq/100 g) and large surface area^46^. Smectite has also been reported to have strong and weak sites in addition to the cation exchange sites such as those in illite^47^. Thus, smectite may be capable of sorbing more REEs than kaolinite and halloysite do at low concentrations.

For illite and smectite, sorption experiments of cations have been relatively well conducted^40,41,47^. However, the chemical states of REEs in individual clay minerals are still unclear at low concentrations. Moldveanu et al.^5–7^ conducted desorption experiments for the bulk of the ion-adsorption ores. The effects of liquid type, ionic strength, pH, and liquid–solid ratio have been evaluated in their experiments. However, desorption experiments for individual minerals are rarely conducted at low concentrations. Understanding the detailed chemical states of individual minerals can aid in suggesting a more efficient extraction method. For instance, if the sorption states at the strong sites were known, it could be possible to improve the extraction ratios of REEs from the ion-adsorption ores.

It has been suggested that soil pH affects the formation of REE concentrated layers^8,36,40^. Around the surface of the soil, the decrease in pH due to the presence of dissolved CO_2_, fulvic and humic acids, which form complexes with REEs^48^, will suppress the sorption of REEs onto the minerals. Conversely, considering the variation of REE concentration in the minerals, mineral composition in the weathered granite, and particularly the presence of illite, presumably contributes to the formation of the REE concentrated layers. The occurrence of illite-rich ion-adsorption ores has not been reported till date^4,8,22^. In this study, the results of XRD and TEM analyses suggested that the amount of illite is small. However, even minor amounts of illite presumably contributes to the formation of ion-adsorption ores due to the high adsorption ability of REEs. Illite is produced during the weathering process of K-feldspar or muscovite to kaolinite^14,15^. Thus, it can be considered that the parent granite rocks of the ion-adsorption deposits commonly form some amount of illite through weathering. Since the peak of illite at 10 Å overlaps that of biotite or muscovite in XRD measurements, it is concerned that the abundance of illite in the ion-adsorption ores has not been accurately assessed in the previous researches. As seen in this study, the amount of illite is often smaller than that of kaolinite or halloysite in weathered granite. Considering their mass balances and sorption abilities for REEs, their contributions for the REEs accumulation should be investigated respectively in the further research. If the parent rock is biotite granite, the contribution of micaceous particles to the formation of ion-adsorption ores increases. Vermiculite is produced during the weathering process of biotite to kaolinite^31^. The degree of weathering from the parent granite presumably influences the formation of the REE concentrated layers. Kaolinite and halloysite become dominant among the clay minerals in the upper layer, which is the late stage of weathering. Illite and vermiculite can be relatively abundant in the REE-enriched second and third layers. The formation of the REE-enriched layer is presumably related to both the change in pH value and the presence of illite and vermiculite.

## Methods

### Weathered granite sample of an ion-adsorption ore

The weathered granite sample used in our study was collected from an ion-adsorption ore in Dingnan County, Jiangxi Province, China^33^. The locality of the weathered granite sample is in the Early Yanshanian granite area, where the major REE deposits are distributed. The thickness of the weathering crust is approximately 10 m. We investigated one of weathered granite samples (ion-adsorption ores) described as 1130S4 in Murakami and Ishihara^33^, whose REE concentration is 474 ppm (Supplementary Table S1). The parent rock of this deposit consists of calc-alkaline granite and alkali granite mainly composed of quartz, K-feldspar, plagioclase, and biotite with minor muscovite and hornblende. In the extraction experiments using 0.5 M ammonium sulfate, 52.9% of REEs were extracted from the weathered granite^9^. In the experiments, HREEs (64.4%) desorbed better than LREEs (51.6%).

### Analytical methods for the weathered granite

The weathered granite sample was dried in an oven at 60 °C and ground in an agate mortar. XRD measurements were conducted on the powdered sample using a Rigaku SmartLab X-ray diffractometer with a D/tex Ultra detector at the National Institute of Advanced Industrial Science and Technology (AIST, Tsukuba, Japan).

Polished sections of weathered granite fixed with epoxy resin were also prepared for the following analyses. Using the polished sections, mineral particles of the weathered granite were analyzed by SEM (JEM-6610LV, JEOL) equipped with EDS (X-MAX, Oxford Instruments). The SEM-EDS analyses were performed at an accelerating voltage of 15–20 kV.

The REE concentration in the individual mineral particles were measured by a quadrupole ICP-MS system (Agilent 7500cx, Agilent Technologies Ltd.) equipped with in-house femtosecond laser-ablation system, incorporates a TiS femtosecond laser (IFRIT, Cyber Laser Inc.) as a 260 nm UV light source by third harmonic generation with a fluence of ~ 30 J/cm^2^ and a pit diameter of ~ 10 μm. Based on the normalization strategy of bulk oxide components as 100 wt%, ten spots were measured to calculate the mean concentration values of REEs. Elemental mapping of a mineral particle was also conducted using time-resolved analysis (TRA) mode by LA-ICP-MS. Speed and density of laser scanning was 10 μm/s and 10 μm/line, respectively. To draw the elemental images, TRA signals were processed by a software called iQuant2^+^^49^.

For in-situ desorption experiments of REEs, the polished sections were immersed in 0.5 M ammonium sulfate solution for 24 h. The pH of the liquid was 5.8. Before and after the treatment, concentrations of Y and REEs in the mineral particle were measured by LA-ICP-MS to calculate in-situ desorption ratio.

From the mineral particles on the polished sections, thin specimens were processed with Ga sputtering using a FIB (JIB-4000), fixed to a cupper grid using micro-manipulator system. For the thin specimens, TEM, HAADF-STEM, and EDS analyses were performed using a JEM 2100F at 200 kV. JIB-4000 and JEM 2100F are operated at the National Institute of Materials and Sciences (NIMS) microstructural characterization platform.

Elemental imaging analyses were performed on FIB sections utilizing the JAMSTEC NanoSIMS 50L at Kochi Institute for Core Sample Research, JAMSTEC. The sections were coated with > 20 nm of Au to mitigate electrostatic charging during analysis. A focused 16 keV O-primary ion beam is scanned across 20 × 20–28 × 28 µm^2^ fields of view depending on the size of FIB section. Positive secondary ions of ^30^Si, ^39^K, ^89^Y, ^138^La and ^147^Sm were measured in multidetection mode with five electron multipliers at a mass resolving power of ~ 3000. We used standard glasses of GM1 (La: approximately 400 ppm), GM5 (Sm: approximately 610 ppm) and Y-Al-garnet to identify target masses for each detector. Details of GM1 and GM5 standard glasses were published in Ito and Messenger^50^. Each run repeatedly scanned (10–25 times) over the same area. Individual images consist of 128 × 128 pixels with acquisition time of 15 ms/pixel (245.76 s/frame). Each measurement was started after stabilization of the secondary ion intensities following a pre-sputtering procedure of approximately 2–5 min.

## Supplementary information

Supplementary Information.
