# Supplementary material for: Microscopic analyses of weathered granite in ion-adsorption rare earth deposit of Jianxi Province, China
Source: Sci Rep. 2020 Nov 19;10:20194. doi: 10.1038/s41598-020-76981-8 (PMC7678834; doi:10.1038/s41598-020-76981-8)
Supplement: Supplementary file 1 — Supplementary Information. [file 41598_2020_76981_MOESM1_ESM.docx]

Supporting Information for

Microscopic analyses of weathered granite

in ion-adsorption rare earth deposit of Jianxi Province, China

Hiroki Mukai^1, 2*^, Yoshiaki Kon^1^, Kenzo Sanematsu^1^, Yoshio Takahashi^3^ & Motoo Ito^4^

^1^National Institute of Advanced Industrial Science and Technology, 1-1-1 Higashi, Tsukuba, Ibaraki, 305-8567, Japan

^2^ Faculty of Life and Environmental Sciences, University of Tsukuba, Tennodai 1-1-1, Tsukuba, Ibaraki 305-8572, Japan

^3^Department of Earth and Planetary Science, Graduate School of Sciences, The University of Tokyo, 7-3-1 Hongo, Bunkyo-ku, Tokyo 113-0033, Japan

^4^ Kochi Institute for Core Sample Research, Japan Agency for Marine-Earth Science and Technology (JAMSTEC), Monobe B200, Nankoku, Kochi 783-8502, Japan

*Corresponding author: Hiroki Mukai

Present address: Faculty of Life and Environmental Sciences, University of Tsukuba, Tennodai 1-1-1, Tsukuba, Ibaraki 305-8572, Japan

Tel: +81-29-853-4012

Email: h-mukai@geol.tsukuba.ac.jp


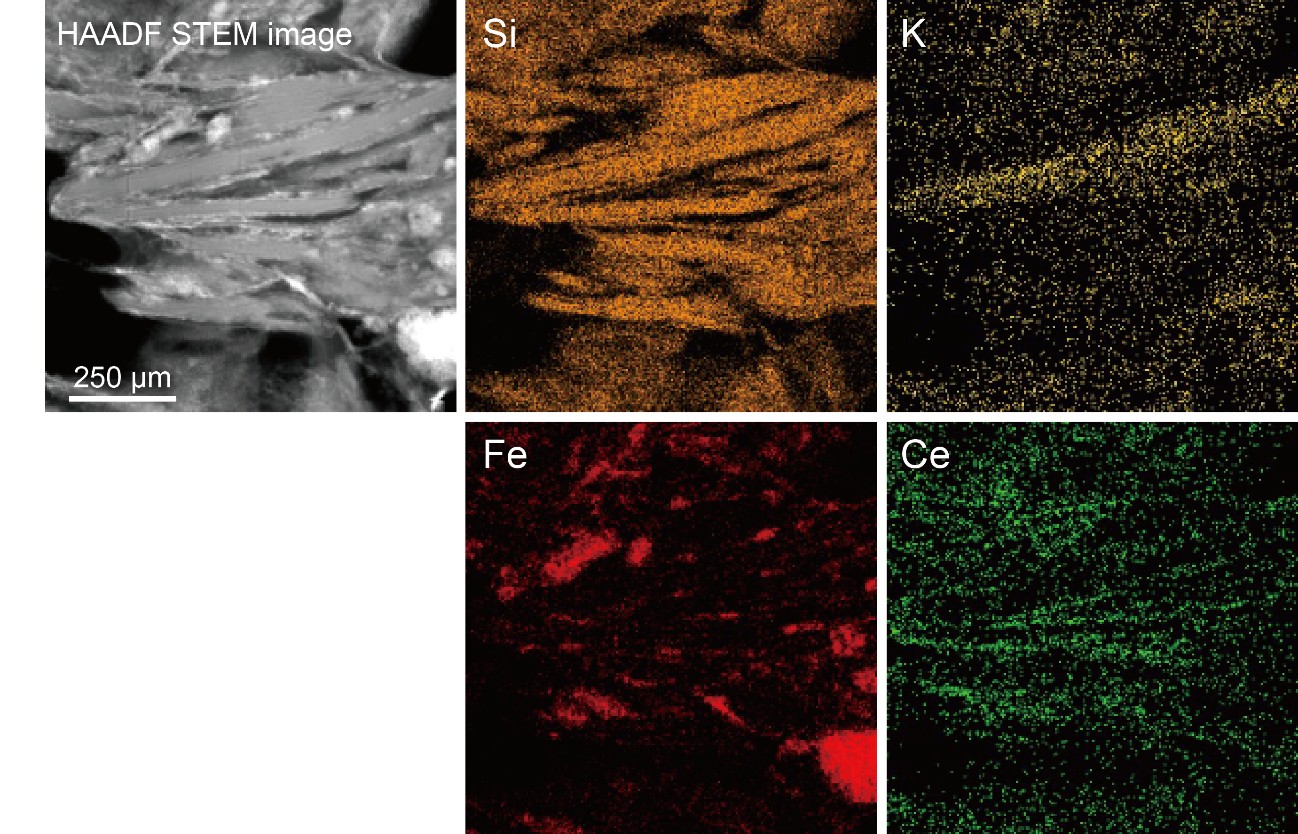


**Supplementary Figure 1: Results of the analyses of the kaolinitic particles by transmission electron microscopy (TEM).** High-angle annular dark field (HAADF)-scanning transmission electron microscopy (STEM) image of a kaolinitic particle, and STEM- energy dispersive X-ray spectrometry (EDS) elemental maps of Si, K, Fe, and Ce.

**Supplementary Table 1**

The concentrations of Y and REEs in the bulk of ion-adsorption ore used in the present study^33^.
